# Virtual reality skateboarding training for balance and functional performance in degenerative lumbar spine disease

**DOI:** 10.1186/s12984-024-01357-2

**Published:** 2024-05-09

**Authors:** Yi-Ching Tsai, Wei-Li Hsu, Phunsuk Kantha, Po-Jung Chen, Dar-Ming Lai

**Affiliations:** 1 School and Graduate Institute of Physical Therapy, College of Medicine, National Taiwan University, 3F., No. 17, Xuzhou Rd., Zhongzheng Dist, Taipei, Taiwan; 2 Physical Therapy Center, National Taiwan University Hospital, Taipei, Taiwan; 3 Faculty of Physical Therapy, Mahidol University, Nakhon Pathom, Thailand; 4 Division of Neurosurgery, Department of Surgery, National Taiwan University Hospital, Taipei, Taiwan

**Keywords:** Virtual reality, Skateboarding, Postural balance, Muscle force, Degenerative lumbar spine disease, Rehabilitation

## Abstract

**Background:**

Degenerative lumbar spine disease (DLD) is a prevalent condition in middle-aged and elderly individuals. DLD frequently results in pain, muscle weakness, and motor impairment, which affect postural stability and functional performance in daily activities. Simulated skateboarding training could enable patients with DLD to engage in exercise with less pain and focus on single-leg weight-bearing. The purpose of this study was to investigate the effects of virtual reality (VR) skateboarding training on balance and functional performance in patients with DLD.

**Methods:**

Fourteen patients with DLD and 21 age-matched healthy individuals completed a 6-week program of VR skateboarding training. The motion capture and force platform systems were synchronized to collect data during a single-leg stance test (SLST). Musculoskeletal simulation was utilized to calculate muscle force based on the data. Four functional performance tests were conducted to evaluate the improvement after the training. A Visual Analogue Scale (VAS) was also employed for pain assessment.

**Results:**

After the training, pain intensity significantly decreased in patients with DLD (*p* = 0.024). Before the training, patients with DLD took longer than healthy individuals on the five times sit-to-stand test (*p* = 0.024). After the training, no significant between-group differences were observed in any of the functional performance tests (*p* > 0.05). In balance, patients with DLD were similar to healthy individuals after the training, except that the mean frequency (*p* = 0.014) was higher. Patients with DLD initially had higher biceps femoris force demands (*p* = 0.028) but shifted to increased gluteus maximus demand after the training (*p* = 0.037). Gluteus medius strength significantly improved in patients with DLD (*p* = 0.039), while healthy individuals showed consistent muscle force (*p* > 0.05).

**Conclusion:**

This is the first study to apply the novel VR skateboarding training to patients with DLD. VR skateboarding training enabled patients with DLD to achieve the training effects in a posture that relieves lumbar spine pressure. The results also emphasized the significant benefits to patients with DLD, such as reduced pain, enhanced balance, and improved muscle performance.

## Background

Degenerative lumbar spine disease (DLD) is a common musculoskeletal disorder in older adults [1]. DLD represents a progressive degenerative condition encompassing spondylolisthesis, lumbar spinal stenosis, and disc degeneration [2]. Globally, an estimated 2.66 billion individuals are affected by DLD [3, 4]. DLD typically presents with various clinical symptoms such as lower back pain, muscle weakness, and paresthesia, which result from nerve root compression [2, 3]. Moreover, balance impairments have been identified in 40–65% of patients with DLD [5, 6] and correlate with walking difficulties and disability. Additionally, patients with DLD often score lower in balance assessments and are at increased risk of falls [7]. All these factors significantly impact the quality of life of patients with DLD [8].

Maintaining balance in daily activities requires the integration of sensory inputs from the visual, vestibular, and somatosensory systems, as well as motor outputs from the musculoskeletal system [9–11]. Patients with DLD display a reduced sense of body position, possibly from abnormalities in paraspinal muscle spindle afference and the processing of central sensory inputs [12]. Research indicates that impaired lumbosacral proprioception contributes to the decline in upright balance among patients with DLD [12, 13]. This altered balance control leads to an increased reliance on ankle proprioception [13]. Additionally, changes in biomechanical characteristics, such as increased sway in the center of pressure (CoP), correlate with compromised balance and suboptimal performance in daily activities [14, 15]. Restricting pelvic movement may serve as a compensatory mechanism for spinal instability and slow walking [6, 14, 16, 17]. Consequently, a thoughtfully designed exercise training program targeting improved balance recovery is imperative for enhancing the quality of life of patients with DLD [18].

The “shopping cart sign,” resembling the action of holding a shopping cart handle, indicates an expanded space in the spinal canal due to the forward flexion posture adopted by patients with DLD. This posture provides significant relief from nerve root compression and associated pain during movement [19]. Additionally, research has highlighted the advantages of incorporating trunk and core muscle group strengthening training in the rehabilitation of patients with chronic lower back pain, leading to improvements in both rehabilitation and balance [20, 21]. Hence, exercise training programs tailored to patients with DLD should integrate these essential elements.

The skateboard exercise training designed in this study, combined with supporting handrails, corresponds to the previously identified movement elements suitable for patients with DLD. Skateboarding also improves coordination and balance, for it requires synchronous engagement of the entire body with muscular and visual input [22]. Furthermore, unlike walking, skateboarding enhances balance compared to walking by promoting greater trunk and hip flexion, activating the knee extensor muscles, and shifting weight onto the supporting leg [23]. As a result, the primary benefits of skateboard training for patients with DLD include (1) engagement in exercise training in a less painful posture, and (2) a focus on single-leg weight-bearing.

Virtual Reality (VR) integrates multisensory stimuli, including visual, auditory, and somatosensory systems [24]. It also enhances participants’ motivation, engagement, and attention, so it is finding wide use in clinical applications. Furthermore, related studies have demonstrated that VR can improve pain management and enhance muscle and balance performance [23, 25–29].

The design of VR skateboarding training [23] not only incorporates unilateral limb training but also theoretically aligns with the activity needs of patients with DLD. We hypothesized that the balance and muscle performance of patients with DLD could be improved through this novel VR skateboarding training. Nevertheless, this novel approach to exercise training has not been applied to individuals with Degenerative Lumbar Disease (DLD). This study sought to assess the impact of exercise training on DLD patients by conducting a comparative analysis with healthy individuals of the same age group.

## Methods

### Participants

A pretest–posttest experimental design was employed in this study to compare the effects of the exercise training on patients with DLD to those in an age-matched group of healthy individuals. The study included 14 participants in the DLD group and 21 participants in the healthy control (HC) group. Table 1 outlines the criteria for both inclusion and exclusion of participants. Every participant gave their informed consent, and the National Taiwan University Hospital’s Institutional Review Board granted approval for the study (Unique Protocol ID: 202003149RINC) and registered at ClinicalTrials.gov (ID: NCT05375201).

**Table 1:** Inclusion and exclusion criteria

	DLD group	HC group
**Inclusion criteria**	(1) A confirmed diagnosis of DLSD based on imaging. (2) Age between 50 and 80 years. (3) Capability to stand and walk independently for at least 5 min.	(1) Absence of any symptoms such as back pain, muscle weakness, or numbness. (2) Age between 50 and 80 years.
**Exclusion criteria**	(1) A history of prior lumbar surgery. (2) Diagnosis of metabolic diseases such as diabetes mellitus. (3) Suffering from vestibular diseases like Meniere’s disease. (4) Presence of neurological disorders like stroke or spinal cord injuries.	

DLD group, patients diagnosed with degenerative lumbar spine disease; HC group, age-matched healthy control group

### VR skateboarding system

**Fig. 1:**
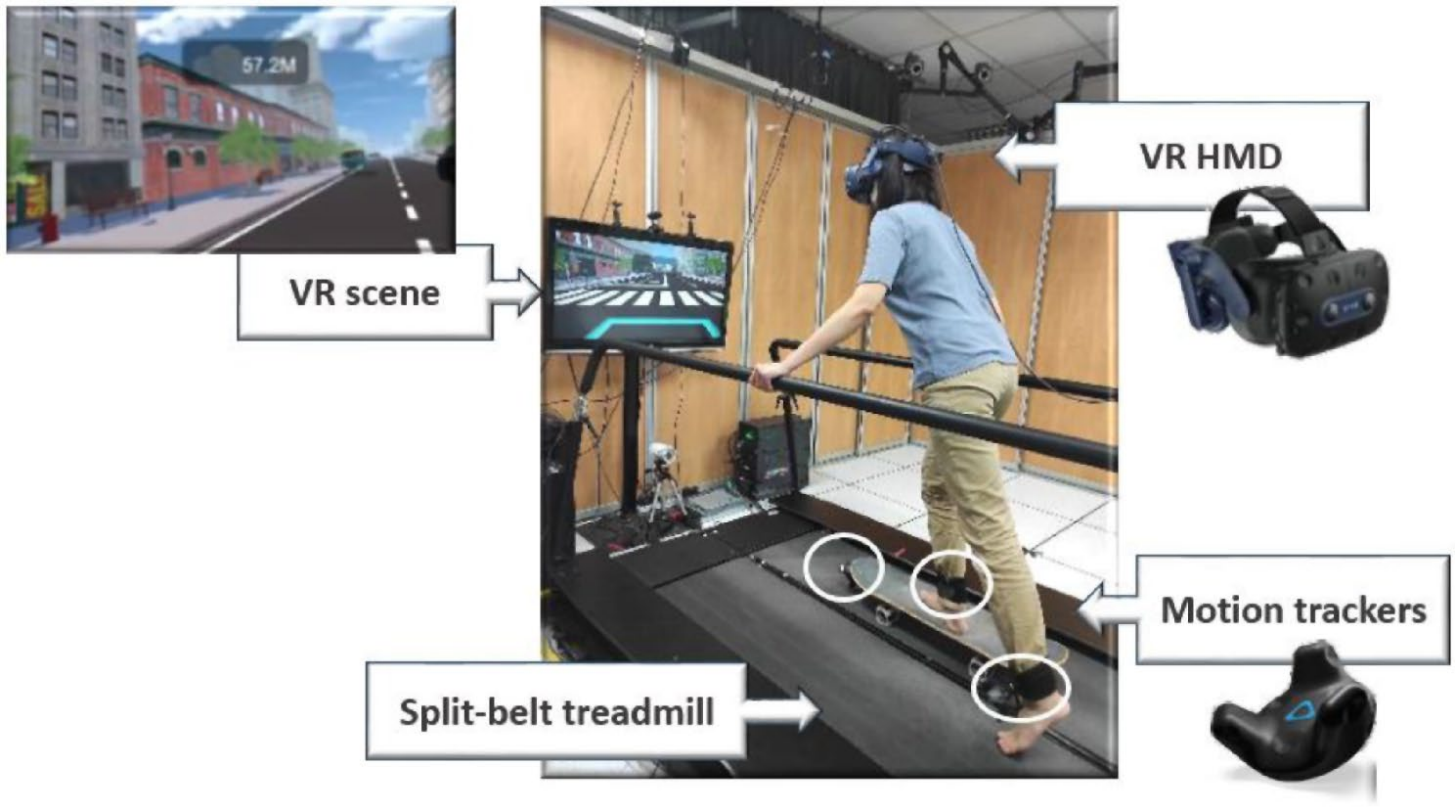
The experimental setup of the VR skateboarding system

VR technology was utilized by integrating Unity3D software (version 5.3.2, San Francisco, USA), a VR head-mounted display (VR HMD), and motion trackers (HTC VIVE, HTC Corporation, New Taipei City, Taiwan). The VR setup was also combined with a split-belt treadmill (QQ-mill, Motekforce Link, Netherlands) and simulated an urban street environment. The skateboard was positioned on the non-moving part of the treadmill, and the moving belt’s speed was set to a pace comfortable for the user (Fig. 1). The wheels of the skateboard were securely immobilized on the treadmill’s stationary belt for safety reasons, and waist-level handrails were provided for support during the VR skateboarding training. This training followed the design proposed in a previous study [23].

### Procedure and training program

**Fig. 2:**
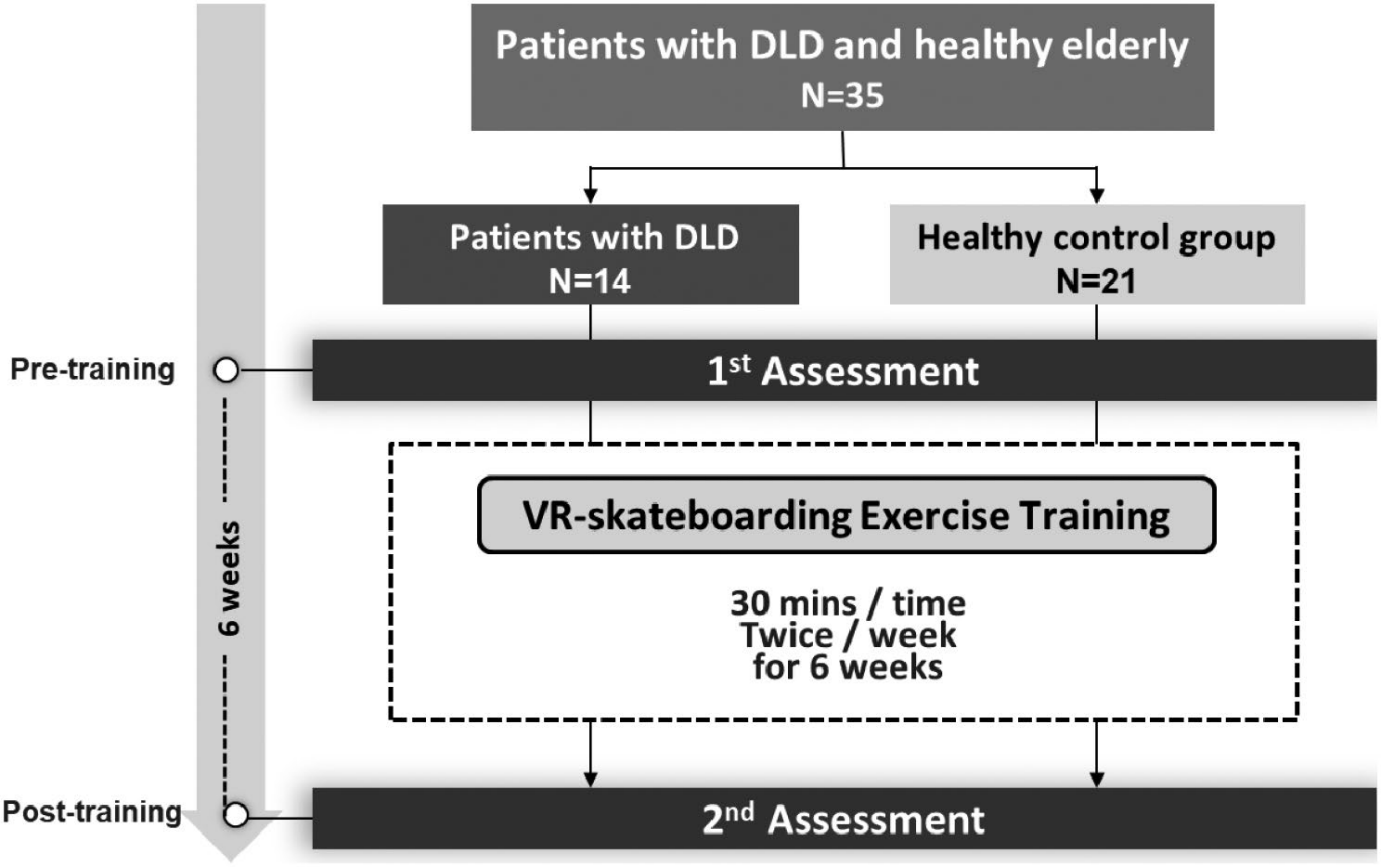
The flowchart of the study protocol

The study protocol is depicted in Fig. 2. Both groups participated in a 6-week training session [23, 30], with assessments one week before and after the training. Participants slid one leg at a comfortable speed on the moving belt [31] while maintaining the other leg on the skateboard for one minute before switching legs. Intervals of rest were provided between leg switching. Each leg was trained for ten repetitions. Hence, the complete VR-skateboard training session lasted about 30 min.

### Assessments and data analysis

The collected data included demographic information and assessments of both functional and balance performance. In addition, for patients with DLD, we utilized the Visual Analogue Scale (VAS) to assess pain intensity from the patient’s perspective [32]. Evaluating the minimum clinically important difference (MCID) achievement provides a more reliable and valid method, assessed by comparing VAS pre-training and post-training differences with established values in relevant literature (MCID of VAS = 2.00) [33].

#### Functional performance assessments

The functional performance assessments included the 10-meter walk test (10MWT), Six-minute walk test (6MWT), Five times sit-to-stand test (5STS), and Timed up and go test (TUG) [34–37]. These tests were used to measure walking speed (m/s), mobility (distance: meters), lower extremity muscle power (duration: seconds), and fall risk (duration: seconds), respectively. Additionally, MCID attainment for functional performance assessments was assessed by comparing pre-training and post-training differences within each group to established literature values (10MWT, MCID = 0.15; 6MWT, MCID = 0.10; 5STS, MCID = 3.70; and TUG, MCID = 2.10) [38–40].

#### Balance performance assessments

As an assessment of postural balance performance, we used the frequently utilized single-leg stance test (SLST) (duration: 30 s) [27]. A 3D motion capture system (120 Hz; Vicon ver. 2.5, Oxford Metrics Ltd., OX, UK) and a force platform (960 Hz; AMTI OR6, Advanced Medical Technology Inc., MA, USA) were employed synchronously to obtain biomechanical characteristics as kinematic and kinetic data for the subsequent analysis of CoP and simulation of muscle force. The data were filtered with a second-order low-pass Butterworth filter with a cutoff frequency of 10 Hz [41] using a custom program developed in MATLAB R2020a software (MathWorks, Natick, MA, USA).

The data from the force platform were used to calculate the CoP in the x-axis (cm) (Eq. 1) and y-axis (cm) (Eq. 2), as well as the resultant CoP (cm) (Eq. 3) [42–44].

1 $$\mathbf{C}\mathbf{o}{\mathbf{P}}_{\mathbf{x}}=\frac{{\mathbf{M}\mathbf{o}\mathbf{m}\mathbf{e}\mathbf{n}\mathbf{t}}_{\mathbf{y}}{+(\mathbf{F}\mathbf{o}\mathbf{r}\mathbf{c}\mathbf{e}}_{\mathbf{x}}\times {\mathbf{D}\mathbf{i}\mathbf{s}\mathbf{t}\mathbf{a}\mathbf{n}\mathbf{c}\mathbf{e}}_{\mathbf{z}})}{{\mathbf{F}\mathbf{o}\mathbf{r}\mathbf{c}\mathbf{e}}_{\mathbf{z}}}$$

where CoP_x_ is the center of pressure on the x-axis, Moment_y_ is the moment on the y-axis, Force_x_ is the force on the x-axis, Distance_z_ is to the distance on the z-axis, and Force_z_ is the force on the z-axis.

2 $$\mathbf{C}\mathbf{o}{\mathbf{P}}_{\mathbf{y}}=\frac{{\mathbf{M}\mathbf{o}\mathbf{m}\mathbf{e}\mathbf{n}\mathbf{t}}_{\mathbf{x}}{+(\mathbf{F}\mathbf{o}\mathbf{r}\mathbf{c}\mathbf{e}}_{\mathbf{y}}\times {\mathbf{D}\mathbf{i}\mathbf{s}\mathbf{t}\mathbf{a}\mathbf{n}\mathbf{c}\mathbf{e}}_{\mathbf{z}})}{{\mathbf{F}\mathbf{o}\mathbf{r}\mathbf{c}\mathbf{e}}_{\mathbf{z}}}$$

where CoP_y_ is the center of pressure on the y-axis, Moment_x_ is the moment on the x-axis, Force_y_ is the force on the y-axis, Distance_z_ is to the distance on the z-axis, and Force_z_ is the force on the z-axis.

3 $$\mathbf{C}\mathbf{o}{\mathbf{P}}_{\mathbf{R}\mathbf{D}}=\sqrt{{\left(\mathbf{C}\mathbf{o}{\mathbf{P}}_{\mathbf{x}}\right)}^{2}+{\left(\mathbf{C}\mathbf{o}{\mathbf{P}}_{\mathbf{y}}\right)}^{2}}$$

where CoP_RD_ is the resultant center of pressure, CoP_x_ is the center of pressure on the x-axis, and CoP_y_ is the center of pressure on the y-axis.

The CoP_x_, CoP_y_, and CoP_RD_ were then used to calculate the following parameters:

1. Mean distance (MDIST) (cm), which is the average distance from the center point, as defined in Eq. 4.

4 $$\mathbf{M}\mathbf{D}\mathbf{I}\mathbf{S}\mathbf{T}=\frac{1}{\mathbf{N}}{\sum }_{\mathbf{n}=1}^{\mathbf{N}}\left|\mathbf{C}\mathbf{o}{\mathbf{P}}_{{\mathbf{R}\mathbf{D}}_{\mathbf{n}}}\right|$$

where MDIST is the mean distance and CoP_RD_ is the resultant center of pressure.

(2) Mean distance in anteroposterior direction (MDIST AP) (cm), which is the average distance from the center point in anteroposterior direction, as defined in Eq. 5.

5 $$\mathbf{M}\mathbf{D}\mathbf{I}\mathbf{S}\mathbf{T} \mathbf{A}\mathbf{P}=\frac{1}{\mathbf{N}}{\sum }_{\mathbf{n}=1}^{\mathbf{N}}\left|\mathbf{C}\mathbf{o}{\mathbf{P}}_{{\mathbf{x}}_{\mathbf{n}}}\right|$$

where MDIST AP is the mean distance in anteroposterior direction and CoP_x_ is the center of pressure on the x-axis.

(3) Mean distance in mediolateral direction (MDIST ML) (cm), which is the average distance from the center point in mediolateral direction, as defined in Eq. 6.

6 $$\mathbf{M}\mathbf{D}\mathbf{I}\mathbf{S}\mathbf{T} \mathbf{M}\mathbf{L}=\frac{1}{\mathbf{N}}{\sum }_{\mathbf{n}=1}^{\mathbf{N}}\left|\mathbf{C}\mathbf{o}{\mathbf{P}}_{{\varvec{y}}_{\mathbf{n}}}\right|$$

where MDIST ML is the mean distance in mediolateral direction and CoP_y_ is the center of pressure on the y-axis.

(4) Mean velocity (MVELO) (cm/sec), which is the postural sway velocity, as defined in Eq. 7.

7 $$\mathbf{M}\mathbf{V}\mathbf{E}\mathbf{L}\mathbf{O} =\frac{\mathbf{T}\mathbf{o}\mathbf{t}\mathbf{a}\mathbf{l} \mathbf{E}\mathbf{x}\mathbf{c}\mathbf{u}\mathbf{r}\mathbf{s}\mathbf{i}\mathbf{o}\mathbf{n}}{\mathbf{T}\mathbf{o}\mathbf{t}\mathbf{a}\mathbf{l} \mathbf{T}\mathbf{i}\mathbf{m}\mathbf{e}}$$

where MVELO is the mean velocity of the resultant center of pressure, Total Excursion is the summation of the resultant center of pressure trajectory, and Total Time is the duration of the postural balance test.

(5) mean frequency (MFREQ) (Hz), which is the postural oscillation, as defined in Eq. 8.

8 $$\mathbf{M}\mathbf{F}\mathbf{R}\mathbf{E}\mathbf{Q} =\frac{{\sum }_{\mathbf{i}=0}^{\mathbf{N}}{\mathbf{f}}_{\mathbf{i}}\times {\mathbf{P}}_{\mathbf{i}}}{{\sum }_{\mathbf{i}=0}^{\mathbf{N}}{\mathbf{P}}_{\mathbf{i}}}$$

where MFREQ is the mean frequency of the resultant center of pressure, f_i_ is the frequency at each interval of the resultant center of pressure trajectory, and P_i_ is the corresponding power of the signal at that frequency interval.

(6) 95% confidence ellipse area (AREA-CE) (cm^2^), which is the postural sway ellipse area with 95% confidence intervals, as defined in Eq. 9.

9 $$\mathbf{A}\mathbf{R}\mathbf{E}\mathbf{A}-\mathbf{C}\mathbf{E}={\uppi }\times \mathbf{M}\mathbf{D}\mathbf{I}\mathbf{S}\mathbf{T} \mathbf{A}\mathbf{P}\times \mathbf{M}\mathbf{D}\mathbf{I}\mathbf{S}\mathbf{T} \mathbf{M}\mathbf{L}$$

where AREA-CE is the 95% confidence ellipse area of the postural balance test, π is pi, MDIST AP is the mean distance in anteroposterior direction, and MDIST ML is the mean distance in mediolateral direction.

**Fig. 3:**
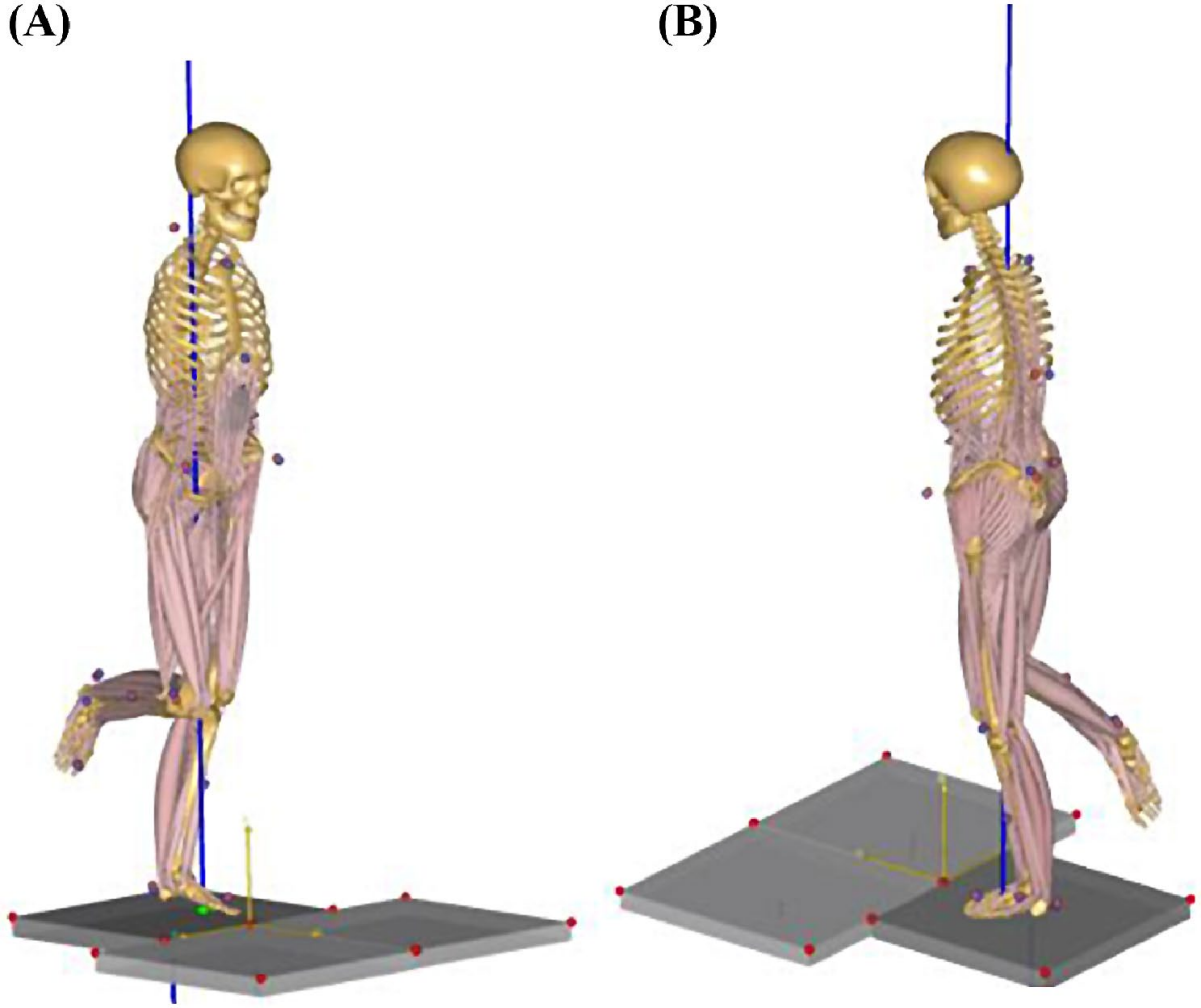
The musculoskeletal simulation of one patient diagnosed with degenerative lumbar spine disease during left single-leg stance task. (A) Anterior view and (B) Posterior view

F Furthermore, as shown in Fig. 3, the marker trajectory and raw data from the force plate were exported to the musculoskeletal simulation system (AnyBody modeling system ver. 7.4.2, AnyBody Technology A/S, A, Denmark) for muscle force computation. The musculoskeletal model employed in this study is accessible in the AnyBody Managed Model Repository (AMMR) provided by AnyBody, with the specific use of the Twente Lower Extremity Model (TLEM-version 2.1) [45]. This musculoskeletal model was scaled and implemented following the data processing protocol outlined in previous studies [46, 47]. Following this, the forces exerted by the activated muscles were calculated and exported, resulting in the acquisition of the normalized mean muscle forces (N/kg) for the gluteus maximus/medius, rectus femoris, biceps femoris, tibialis anterior, and gastrocnemius.

### Statistical analysis

SPSS version 23.0 software (SPSS Inc., Chicago, IL, USA) was used for statistical analyses, with a significance α level of 0.05. The normality of all variables was evaluated using the Shapiro-Wilk test, which revealed non-normal distribution. In the SLST test, the Wilcoxon signed-rank test was used to determine whether there were differences between the left and right foot. Consequently, comparisons of all variables between groups before and after the training were conducted using the Mann-Whitney U test. The Wilcoxon signed-rank test was used to examine changes within each group from pre-training to post-training.

## Results

A total of 14 participants diagnosed with DLD (6 males and 8 females) and 21 healthy control participants (5 males and 16 females) were enrolled. Table 2 shows demographic information for both groups. The VAS scores for the DLD group before and after the training were 4.93 ± 1.54 and 2.14 ± 2.03, respectively, showing a significant decrease in pain scores after the training (*p* = 0.024). Additionally, 78.57% of participants in the DLD group achieved the MCID for VAS [33]. This indicates an improvement in pain intensity after the exercise training.

**Table 2:** Demographic information

	DLD group	HC group	p value
**No. of participants**	14	21	
**Sex (male/female)**	6/8	5/16	0.241
**Age (years)**	64.14 ± 7.81	62.62 ± 6.98	0.543
**Height (cm)**	163.31 ± 8.88	160.38 ± 6.81	0.345
**Weight (kg)**	64.87 ± 14.48	62.33 ± 10.54	0.613
**Body mass index (kg/m** ^**2**^ **)**	24.22 ± 1.45	24.14 ± 3.08	0.814
**Leg dominant (left/right)**	0/14	0/21	

Values are presented as mean ± standard deviation or numbers. DLD group, patients diagnosed with degenerative lumbar spine disease; HC group, age-matched healthy control group

### Functional performance assessments

**Fig. 4:**
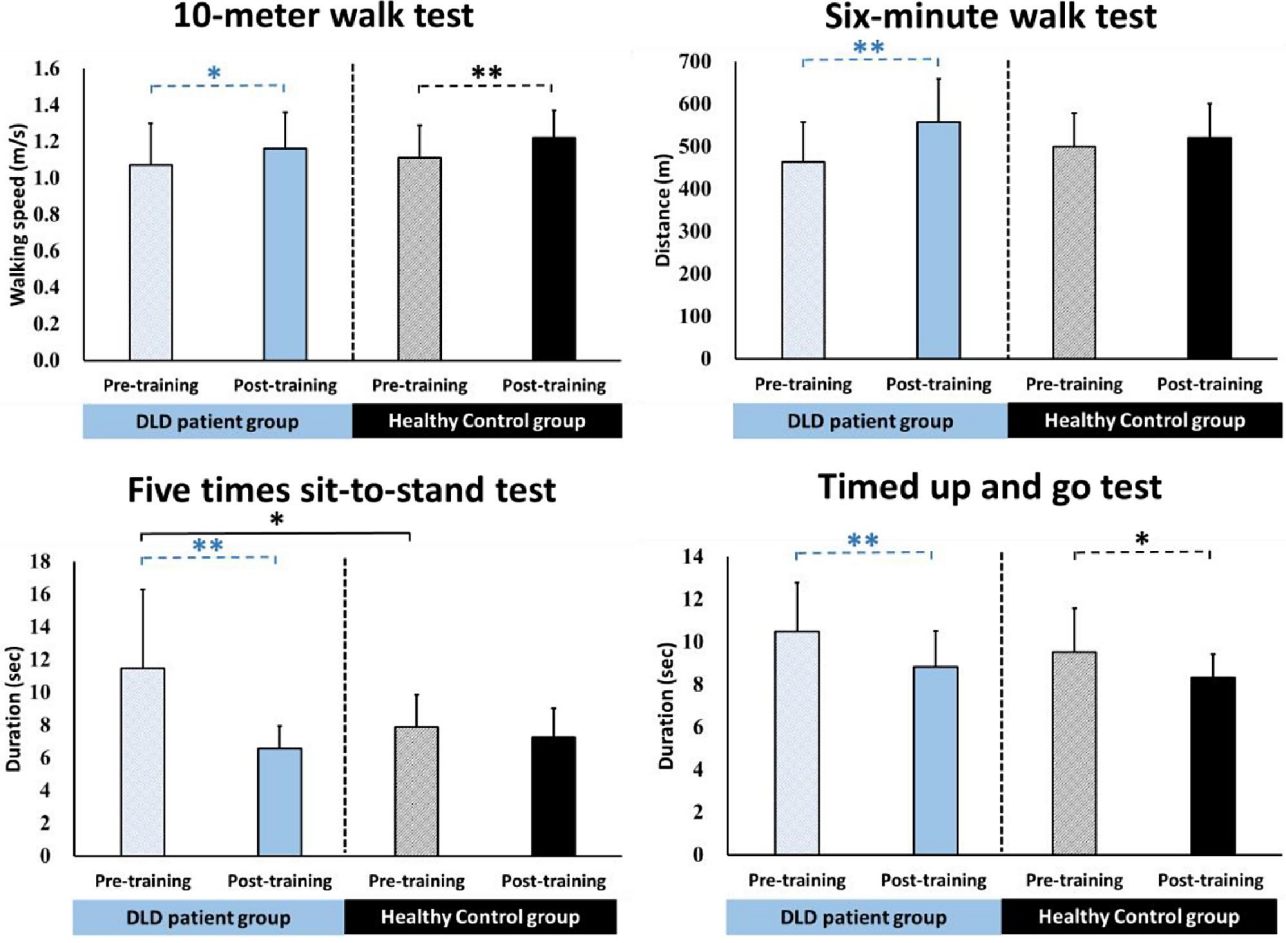
The results of functional performance assessments. Values are presented as mean ± standard deviation. ^*^, *p* < 0.05; ^**^, *p* < 0.01

The results of the 10MWT, 6MWT, 5STS, and TUG are presented in Fig. 4. Before the training, the DLD group required more time to complete the 5STS test, exhibiting significantly worse performance compared to the HC group (*p* = 0.024). After the training, however, no significant differences were observed in any of the functional assessments between the two groups (*p* > 0.05). This suggests that the performances of the DLD and HC groups were similar after the training. When examining within-group comparisons, the DLD group exhibited significant improvements in all functional assessments after the training (10MWT, *p* = 0.010; 6MWT, *p* = 0.008; 5STS, *p* = 0.001; and TUG, *p* = 0.001). Additionally, in the DLD group, the proportions of participants achieving the MCID in the four functional assessments were as follows: 10MWT: 35.71%; 6MWT: 71.43%; 5STS: 50.00%; and TUG: 42.86% [38–40]. In contrast, the HC group showed significant improvements only in the 10MWT and TUG tests after the training (*p* = 0.006 and 0.011, respectively). Furthermore, in the HC group, the proportions of participants achieving the MCID in the four functional assessments were as follows: 10MWT: 38.10%; 6MWT: 42.86%; 5STS: 9.52%; and TUG: 23.81% [38–40].

### Balance performance assessment

The statistical results showed no significant changes in kinetic parameters between the left and right foot in either group, which is similar to Hoffman et al.‘s [48] findings in healthy individuals. As a result, the data regularly chosen for analysis represented the non-dominant foot (which acted as the supporting foot) as the reference for subsequent between-group and within-group statistical analyses.

#### Kinetic data

The statistical results on CoP are listed in Table 3. In the between-group comparison of CoP, we observed that all parameter values in the DLD group were significantly higher than those in the HC group before the training (*p* < 0.05), except for mean frequency (*p* = 0.014). This observation indicates that the disease had an impact on the balance performance of the DLD group. After the training, there were no significant differences between the two groups in mean distance, mean AP distance, mean ML distance or AREA (*p* = 0.069, 0.706, 0.088 and 0.690, respectively). While a significant difference in mean velocity remained, the value showed a positive trend in the HC group. This suggests that the DLD and HC groups had similar performances in these aspects after the training.

**Table 3:** Center of pressure in supporting leg during single-leg stance test

	DLD group (*N* = 14)		HC group (*N* = 21)		p value			
	Pre-training	Post-training	Pre-training	Post-training	Between-group		Within-group	
					Pre-training	Post-training	DLD group	HC group
**Mean distance (cm)**	0.68 ± 0.14	0.56 ± 0.12	0.53 ± 0.13	0.49 ± 0.11	0.007^**^	0.069	0.003^**^	0.029^*^
**Mean AP distance (cm)**	0.75 ± 0.19	0.56 ± 0.13	0.62 ± 0.26	0.59 ± 0.18	0.033^*^	0.706	0.003^**^	0.670
**Mean ML distance (cm)**	0.64 ± 0.14	0.56 ± 0.10	0.50 ± 0.10	0.49 ± 0.11	0.003^**^	0.088	0.013^*^	0.897
**Mean velocity (cm/sec)**	3.74 ± 1.13	3.18 ± 0.83	2.49 ± 0.63	2.35 ± 0.77	0.003^**^	0.021^*^	0.008^**^	0.201
**Mean frequency (Hz)**	0.61 ± 0.14	0.71 ± 0.13	0.54 ± 0.18	0.57 ± 0.18	0.184	0.014^*^	0.037^*^	0.133
**AREA (cm** ^**2**^ **)**	58.92 ± 26.90	34.70 ± 13.32	35.81 ± 22.94	31.86 ± 12.60	0.006^**^	0.690	0.003^**^	0.407

Values are presented as mean ± standard deviation. DLD group, patients diagnosed with degenerative lumbar spine disease; HC group, age-matched healthy control group; AP, in anteroposterior direction; ML, in mediolateral direction; AREA, 95% confidence ellipse area; ^*^, *p* < 0.05; ^**^, *p* < 0.01

However, there was a significant between-group difference in mean frequency due to an increase in values in the DLD group (*p* = 0.014). In the within-group comparison, apart from the increase in mean frequency values (*p* = 0.037) in the DLD group, all other parameter values significantly decreased after the training (*p* < 0.05). In contrast, the HC group showed significant decreases only in mean distance after the training (*p* = 0.029). The DLD group exhibited greater improvements than did the HC group.

#### Muscle force

The statistical results of normalized muscle force are presented in Table 4. In the between-group comparisons, we found that during the SLST, the DLD group required more muscle force in the biceps femoris to maintain balance than the HC group did (*p* = 0.028), while the performances of other muscle groups exhibited similar patterns before the training. After the training, there were no significant differences between the two groups in parameters (*p* > 0.05), indicating comparable muscle performance in the DLD group. The only exception was that the DLD group exhibited a greater need for gluteus maximus strength to maintain balance during the SLST (*p* = 0.037).

**Table 4:** Muscle force in supporting leg during single-leg stance test

Muscle force (N/Kg)	DLD group (*N* = 14)		HC group (*N* = 21)		p value			
	Pre-training	Post-training	Pre-training	Post-training	Between-group		Within-group	
					Pre-training	Post-training	DLD group	HC group
**Gluteus maximus**	1.63 ± 1.27	1.56 ± 0.84	1.19 ± 1.16	0.91 ± 0.94	0.423	0.037^*^	0.917	0.744
**Gluteus medius**	11.08 ± 2.92	13.50 ± 4.70	12.45 ± 4.69	12.58 ± 4.02	0.447	0.548	0.039^*^	0.845
**Rectus femoris**	0.59 ± 1.16	1.17 ± 2.86	0.56 ± 0.82	0.71 ± 1.31	0.603	0.603	0.345	0.500
**Biceps femoris**	2.47 ± 1.95	2.63 ± 2.67	1.06 ± 1.54	1.09 ± 1.34	0.028^*^	0.230	0.650	0.879
**Tibialis anterior**	0.40 ± 1.08	0.65 ± 2.05	1.77 ± 2.60	1.24 ± 2.03	0.317	0.719	0.814	0.112
**Gastrocnemius**	9.12 ± 6.77	9.23 ± 3.90	12.64 ± 5.67	12.95 ± 6.52	0.078	0.093	0.600	0.647

Values are presented as mean ± standard deviation. DLD group, patients diagnosed with degenerative lumbar spine disease; HC group, age-matched healthy control group; ^*^, *p* < 0.05

In the within-group comparisons, the DLD group showed a significant increase in muscle strength in the gluteus medius (*p* = 0.039), suggesting a slight alteration in muscle force pattern after the training. However, the HC group showed no significant differences in muscle force after the training (*p* > 0.05).

## Discussion

Exercise training that improves balance and muscle performance is essential for patients with DLD. This was the first study to integrate VR and skateboarding for exercise training in patients with DLD. Based on the results of VAS, it can be observed that the majority of DLD patients experienced a reduction in pain after training, reaching the MCID [33]. In addition, we conducted a quantitative analysis of the outcomes to determine the effectiveness of the training.

Before the training, the results of the 5STS indicated that the patients with DLD exhibited lower extremity muscle power than that of healthy individuals. Regarding demographic information, both the DLD and HC groups showed no difference in age. This functional performance assessment requires demands a high level of agility to perform it as quickly as possible [49]. Due to the DLD condition, patients may experience delayed recruitment of deep trunk muscle activation, as well as impaired lower limb power, both of which can impact their performance on the task [50–52]. This indicates that patients with DLD require training to enhance lower limb muscle strength. After training, the leg muscle power of patients improved to the same level as healthy individuals.

The 10MWT and TUG revealed no significant differences, as they only required participants to perform at their usual levels of effort. The 6MWT may be equally challenging for healthy individuals, making it difficult to distinguish differences between the two groups. Therefore, the 5STS test is recommended for clinical comparisons between individuals with and without DLD.

After the training, no significant differences were observed in all functional performance between the two groups, indicating that the patients with DLD, through training, reached the same level as trained healthy individuals. This suggests a positive training outcome. The within-group results also support the aforementioned points. The patients with DLD demonstrated significant improvements in all functional performance, i.e., walking speed, mobility, and lower extremity muscle power, along with a reduced risk of falling. Furthermore, 5STS and 6MWT achieved MCID by half to the majority of participants, further supporting the training’s benefits [38, 39]. In comparison, healthy individuals showed improvements only in walking speed and reducing the risk of falling. This suggests that the intervention had more comprehensive training effects on patients with DLD compared to healthy individuals. Additionally, only a small number of healthy individuals achieved the MCID for each assessment [38–40].

The results of the balance test produced several noteworthy findings related to performance in the SLST with the supporting foot (left foot). Many studies utilized center of pressure characteristics to assess postural stability during quiet standing [53]. Except for mean frequency, all parameters linked to postural sway consistently indicated worse balance performance in the patients with DLD than in healthy individuals, both in the overall trajectory and in distinct directions before the training. Increased sway or displacement of the CoP is associated with impaired balance and functional performance [54]. These findings imply that effective balance training is indeed necessary for patients with DLD.

Mean distance, mean AP distance, mean ML distance, and AREA showed no differences after the training, indicating that the patients with DLD approached the stability of healthy individuals in all directions and overall sway range. This may be attributed to the fact that skateboarding primarily involves dynamic movements in anteroposterior direction, including body propulsion and moving the foot forward or backward, combined with the visual stimuli of VR progression, thus enhancing training in anteroposterior stability. Our previous research findings support greater hip flexion and ankle dorsiflexion [23], which may contribute to forward and backward movement stability. Moreover, the training may require regulation of the left–right movement of the skateboard and contribute to the enhancement of lateral stability.

Considering the results within each group, it is evident that the patients with DLD exhibited improvements in balance in various directions after the training. These changes signified enhancement of their balance ability abilities and supported the presence of positive training benefits. However, the healthy individuals showed quantifiable reductions only in mean distance after the training, indicating that their balance ability remained stable in general. The overall results on CoP positively supported the argument for training benefits, particularly for patients with DLD.

Due to the improvement shown in all parameters but mean frequency, we believe that the patients with DLD may have shifted their balance strategy toward greater reliance on ankle proprioceptive control [13]. This adjustment could be due to the instability experienced while standing on a skateboard, which may tilt unpredictably. The instability of the skateboard could lead patients with DLD to rely more on ankle proprioception to maintain balance. Consequently, the patients with DLD increased the frequency of adjustments to maintain balance within a reduced range of sway. Fast coordination, as suggested by Suzuki et al. [55], is a crucial issue in maintaining a standing balancing task. In this regard, we conclude that the patients with DLD improved their rapid adjustment abilities after the training.

Musculoskeletal modeling is an approach that integrates detailed human trajectories obtained via motion capture techniques to calculate both kinetic data and muscle forces during dynamic functional tasks [56–58]. The AnyBody musculoskeletal simulation has been widely utilized for understanding muscle forces in various activities [46, 47, 56, 59], and using marker data from the kinematic model to drive the musculoskeletal model segments has been validated and recognized for its high reliability and precision [60]. In our study, we further employed musculoskeletal modeling to conduct inverse dynamics analysis to evaluate muscle force performance during the SLST. Comparisons between and within groups revealed similar patterns. However, a notable finding was that patients with DLD required the utilization of more biceps femoris force before the training than did the healthy individuals. Interestingly, after the training, patients with DLD utilized more gluteus maximus to maintain stability compared to healthy individuals. Furthermore, it was observed that the force generated by the gluteus medius increased in patients with DLD after the training.

Our interpretation of these findings is that patients with DLD had inferior balance performance [12, 13] and muscle weakness in the gluteus medius [61] before the training. They needed to employ more biceps femoris force to compensate. However, after the training, there was a shift toward the increased utilization of the gluteus medius and gluteus maximus forces to maintain stability during the SLST. This suggests that the training had an impact on the muscle force distribution and balance in the patients with DLD, thereby increasing the involvement of muscles in the proximal regions of the body.

From a clinical perspective, the results strongly support the benefits of the skateboard training for patients with DLD due to its focus on SLS and hip flexion in a flexed posture with hands supporting. These specific movements could be adjusted to meet the specific needs of patients with DLD, potentially leading to improvements in pain relief, muscle performance, and balance. As future customization can be performed based on the individual needs and expectations of other user patients or groups, the existing VR skateboarding settings may be improved by modifying the training intensity or scenarios to enhance functional performance and balance. While we have demonstrated that this training can maintain or even improve the performance of healthy individuals, it is evident that this group is suitable for more challenging and demanding training.

The most significant challenge in this study was the difficulty in recruitment due to the ongoing COVID-19 pandemic. Another major limitation, as previously mentioned in our published work [23], was that handrails may potentially dilute the true training effects. However, due to safety considerations, especially that of clinical patients with impaired balance function, the use of this setup is still necessary.

## Conclusions

This study demonstrates that patients with DLD exhibited functional and balance deficiencies, emphasizing the necessity for training interventions. It further shows that the novel VR skateboarding exercise training provides substantial advantages to patients with DLD, enhancing their balance and muscular performance. The findings emphasize the importance of tailored training interventions for specific populations to enhance their functional and balance abilities and thereby reduce the need for surgery and their economic burdens while enhancing overall quality of life.

## Data Availability

The data used in this study are available from the corresponding author upon reasonable request.

## Abbreviations

DLD: Degenerative lumbar spine disease
VR: Virtual reality
SLST: single-leg stance test
VAS: Visual analogue scale
CoP: Center of pressure
MCID: Minimum Clinically Important Difference
10MWT: 10-Meter Walk Test
6MWT: Six-Minute Walk Test
5STS: Five times sit-to-stand test
TUG: Timed up and go test
AP: In Anteroposterior Direction
ML: In Mediolateral Direction
AREA: 95% confidence ellipse area
